# Redescription and new records of *Ulomimus indicus* Bates, 1873 (Coleoptera, Tenebrionidae, Tenebrioninae)

**DOI:** 10.3897/zookeys.357.6402

**Published:** 2013-12-02

**Authors:** Shanshan Liu, Guodong Ren, Ottó Merkl

**Affiliations:** 1 College of Life Sciences, Hebei University, Baoding, 071002, P. R. China; 2 Hungarian Natural History Museum, H-1088 Budapest, Baross utca 13, Hungary

**Keywords:** China, Coleoptera, Indonesia, new country records, Tenebrionidae, Thailand, *Ulomimus*, Ulomini

## Abstract

*Ulomimus indicus* Bates, 1873 of the tribe Ulomini is recorded for the first time from China (Guangxi and Hainan), Indonesia (Sumatra) and Thailand (Chiang Dao). A redescription of the male and the first description of the female are also provided.

## Introduction

*Ulomimus indicus* and the monotypic genus *Ulomimus* were described by Bates (1873) from “East India”. The species was later mentioned to occur in Sri Lanka (Kaszab 1979) and in Vietnam (Kaszab 1980, Merkl 1992).

*Ulomimus* is a member of the tribe Ulomini. This tribe is characterized by the presence of placoid sensoria on the antennae, the primitive (“lagrioid”) type of labrum and the exposed posterior part of the 7^th^ abdominal tergite (Matthews and Bouchard 2008). The tribe includes about 40 genera and an estimated 400 described species. It is fairly speciose in the Old and New World tropics, but very few species extend into the Nearctic ecozone (Aalbu et al. 2002) and into the Euro-Siberian region of the Palaearctic ecozone (Löbl et al. 2008). More than half of the known species have been described from the Indomalaya ecozone (Oriental Realm), where Sundaland is the richest in species. However, the overwhelming majority of the described species belong to the genus *Uloma* Dejean, 1821.

*Ulomimus* is very similar and closely related to the Oriental genus *Cneocnemis* Gebien, 1914. In *Ulomimus* the antennomere 6 is transverse and bears one placoid sensorium (antennomere 6 is subequal in length and width and without placoid sensorium in *Cneocnemis*), the pronotum is widest in anterior 1/3 (widest at base in *Cneocnemis*), and apicale of aedeagus with small oblique lateral notch (without notch in *Cneocnemis*).

These two genera are similar to the widely distributed genus *Uloma*. The antennomeres 5 to 10 of *Uloma* are strongly transverse, forming a more or less distinct club, and have several placoid sensoria on their distal edge, which are arranged in complete rings on antennomeres 7 to 10. Antennae of *Cneocnemis* and *Ulomimus* are more elongate, only the antennomeres 6 or 7 to 10 are transverse (much less than in *Uloma*), the placoid sensoria are fewer in number, and do not form complete rings. The pronotum of most *Uloma* species is sexually dimorphic – males have large anterior impression and low protuberances. The pronotum of the males and females are the same in *Cneocnemis* and *Ulomimus*.

In the present paper, the distribution range of the species is revised through the inclusion of new records from China, Indonesia and Thailand. A redescription of the male and a description of the female (including diagnostic features) are also provided.

## Material and methods

The illustrations were made using a Nikon SMZ800 dissecting microscope (equipped with a camera lucida). The photos were taken with a Leica M205A stereomicroscope equipped with a Leica DFC 450 digital microscope camera. All measurements were made in millimeter. The specimens examined are deposited in the Museum of Hebei University (MHBU), Baoding, China, in the Hungarian Natural History Museum, Budapest, Hungary (HNHM) and in the National Museum of Nature and Science, Tokyo (now in the Masumoto Collection, NMNS).

## Taxonomy

### Ulomimus

Bates, 1873

http://species-id.net/wiki/Ulomimus

Ulomimimus Bates, 1873: 201; Neave 1940b: 608.
Ulomimus *Ulomi[mi]mus*: Rye 1873: 288.
Ulomimus : Gebien 1911: 399; Lucas 1920: 665; Gebien 1914: 33; Gebien 1940: 770 [577]; Kaszab 1979: 88; Kaszab 1980: 175; Merkl 1992: 263.
Pseuduloma Fairmaire, 1893: 27; Gebien 1911: 404; Carter 1926: 508; Neave 1940a: 1011.

#### Type species.

*Ulomimus indicus* Bates, 1873.

#### Diagnosis.

Antennae short, antennomeres 6 to 10 gradually widened, 11 large, nearly globose. Pronotum with narrow and complete basal bead. Protarsi dilated with a brush of dense short hairs beneath, and protarsomere 4 much smaller than 2 and 3. Protibia strongly triangularly widened near apex, with large tooth on ventral surface. Aedeagus linearly truncate at apex in dorsal view, parameres with a small notch behind each apical corner.

#### Remarks.

The original spelling of the generic name is *Ulomimimus*. Rye (1873) mentioned the name as *Ulomi[mi]mus*, which is regarded as unjustified emendation. Neave (1940b) considered *Ulomimus* as an “err. Pro *Ulomimimus* Bates, 1873”. However, all other authors used *Ulomimus*, so *Ulomimus* should be an unjustified emendation by Rye (1873) in prevailing usage, which is according to the Art. 33.2.3.1 of the ICZN (1999) is deemed a justified emendation.

It is unknow for the authors of the present paper who synonymised *Pseuduloma* with *Ulomimus*. Carter (1926) mentioned *Pseuduloma* as a distinct genus, when transferred *Alphitobius torridus* Carter, 1911 (now belonging to *Scotoderus* Perroud, 1864, see Matthews and Bouchard 2008) to this genus. Neave (1940a) also considered *Pseuduloma* as a valid genus. However, Gebien (1940) used *Pseuduloma* as a synonym of *Ulomimus*.

### Ulomimus indicus

Bates, 1873

http://species-id.net/wiki/Ulomimus_indicus

Figs 1 –13

Ulomimimus indicus Bates, 1873: 202.
Ulomimus indicus : Gebien 1911: 399; Gebien 1940: 770 [577]; Kaszab 1979: 88; Kaszab 1980: 175; Merkl 1992: 263.
Pseuduloma cribricollis Fairmaire, 1893: 27; Gebien 1911: 404.

#### Original description.

“Oblong, sub–parallel, moderately convex; brownish–black, shining, the mentum, antenna, palpi, tarsi, labrum, and margins of epistoma ferruginous, the legs chestnut–red; head coarsely and closely reticulate–punctate; prothorax punctured–sparsely on the disc–the punctures large, deep, rounded, and partly filled in with an apparent exudation of an ashy tint; scutellum smooth; elytra with nine (including the extreme marginal one) fine but deep striae, and a short scutellar one, the striae punctured (the punctures being much wider than the striae, the elytra appear crenulate–striate), the 4^th^ and 5^th^ striae shortest and united at some distance from the apex; intervals convex posteriorly, very minutely and sparsely punctured; pro– and mesosterna, flanks of pro– and mesothorax, and base of epipleural fold, strongly and closely punctured; metasternum, abdomen, and femora sparingly punctured, abdominal joints rugulose at the base. Long. corp. 4 lin.

Hab.: East India; one example.”

#### Redescription.

**Figures 1–2.**
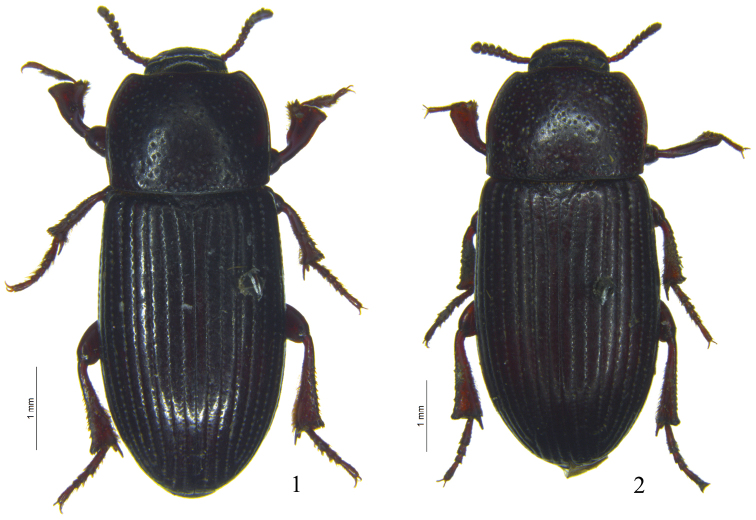
*Ulomimus indicus* Bates, 1873. **1** Male **2** female.

**Figures 3–13.**
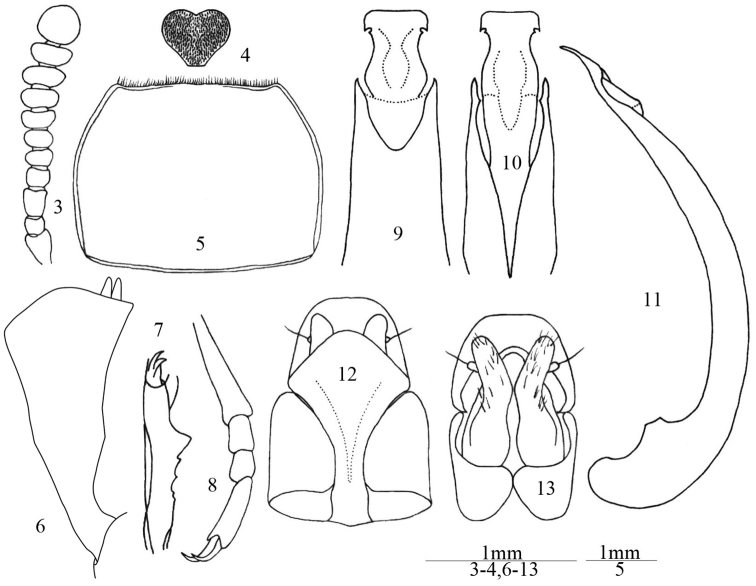
*Ulomimus indicus* Bates, 1873. **3** Antenna, male, dorsal view **4** mentum, male, ventral view **5** pronotum, male, dorsal view **6** protibia, male, dorsal view **7** protibia, male, lateral view **8** metatarsus, male, dorsal view **9** apical aedeagus, dorsal view **10** apical aedeagus, ventral view **11** aedeagus, lateral view **12** ovipositor, dorsal view **13** ovipositor, ventral view.

Male. Body length 8.0–9.0 mm; width 3.5–4.0 mm. Body (Fig. 1) elongate, elliptical, black or dark brown. Antennae, mouthparts and legs slightly paler. Head transverse, with small punctures on anterior half, and with sparse large punctures on posterior half; labrum transversely rectangular, densely punctate, scattered with short and yellow hairs; clypeus densely punctate, anterior margin truncate; frontoclypeal suture deeply impressed; genae feebly convex and slightly extended, temples reduced; eyes transverse, with 5–6 facets at narrowest point in lateral view; frons weakly convex, with large punctures; mentum (Fig. 4) cordate, truncate basally, with short and very dense yellow pubescence; ligula deeply emarginate anteriorly, depressed in middle; maxillary palp with narrowly trapezoidal terminal palpomere. Antennae (Fig. 3) short, not reaching half of pronotum; antennomere 1 thick, 2 very short, 3 long and narrow, 4 and 5 short, 6 to 10 gradually widening, 11 nearly globose, ratio of the length (width) of antennomeres 2–11 as follows: 4 (6): 8 (6): 5 (7): 5 (7): 5 (8): 4 (9): 5 (10): 5 (12): 5 (11): 11 (11). Antennomere 6 with one placoid sensorium on inner anterior corner, 7 with two placoid sensoria on inner corner, 8 to 9 with a few on inner and outer corners.

Pronotum (Fig. 5) transverse, about 1.35 times as wide as long, widest at anterior 1/3, with large punctures widely spaced in middle but becoming denser toward sides; anterior margin emarginate with narrow bead interrupted in middle, and with dense short hair fringes; lateral margins arcuate, strongly narrowing forward and less so from widest point to base, with narrow bead; basal margin slightly convex, with narrow bead; anterior angles nearly rectangular, posterior angles obtuse. Prosternum with sparse and large punctures, prosternal process rounded in lateral view, with small subapical tubercle. Mesoventrite with deep triangular impression; mesepisternum, metepimeron and metepisternum coarsely and sparsely punctate.

Scutellum triangular, impunctate. Elytra distinctly punctato–striate, intervals weakly convex, very finely and sparsely punctate, lateral margins visible only at humeri in dorsal view. Epipleura sparsely and coarsely punctate in basal 1/3.

Protibia (Figs 6–7) with two equal apical spurs; narrow at base, then explanate on both inner and outer edges, outer edge forming blunt subapical angulation, inner edge slightly concave at middle; outer edge without denticulation; inner edge fringed with yellow hairs becoming denser and longer toward apex; dorsal surface with low and blunt longitudinal keel and with fine and sparse punctures; ventral surface with sharp longitudinal keel and sharp tooth at middle (Fig. 7), ventral surface with a few coarse punctures and short, sparse, thick hairs. Protarsomeres 2 to 4 dilated, with long, sparse yellow dorsal hairs and dense, yellow ventral hair pads. Mesotibia and metatibia gradually dilated toward apex, outer edge with small denticles and sparse, long hairs. Length ratio of metatarsomeres 1 to 4 (Fig. 8) as follows: 10: 3: 3: 7.

Abdominal ventrites finely and sparsely punctate, punctuation denser and subcontiguous toward lateral portions; last ventrite with deep apical groove.

Aedeagus (Figs 9–11) with basale parallel–sided; apicale broad at base, constricted at middle, widening and truncate at apex in dorsal view, with longitudinal depression in ventral view, slightly curved in lateral view; with small oblique notch at posterior corners of widened apical part.

Female (Fig. 2). Mentum cordate, without dense pad of pubescence, but with sparse hairs and coarse wrinkles. Protibia with shape similar to or narrower than that of male, ventral surface concave, without keel and large tooth. Protarsomeres 2 to 4 not dilated and without ventral hair pads. Ovipositor (Figs 12–13) with coxites relatively smooth, bearing long sensorial hairs and a few short hairs at base.

#### Examined materials.

1♀ (MHBU): China, Guangxi, Tian’e County, 14 September 2002, M. Bai leg; 1♂, 1♀ (HNHM), 2♂♂, 2♀♀ (MHBU): China, Hainan, Baisha County, Nankai Town, Shenbo Village, 1 June 2007, Y. B. Ba leg; 1♀ (NMNS): Thailand, Chiang Dao Hill Resort, 10–11 November 2012, K. Masumoto & K. Takahashi leg; 1♀ (HNHM): Indonesia, Sumatra, Dolok Merangir, 25 June 1970, collector unknown; 1♂ (HNHM): Indonesia, Sumatra, Palembang, date unknown, W. Knappert leg.

#### Distribution.

“East India” (Bates 1873); Sri Lanka (Kaszab 1979); Vietnam (Fairmaire 1893, Kaszab 1980, Merkl 1992); Thailand, Indonesia/Sumatra, China/Guangxi and Hainan Provinces (new records).

## Supplementary Material

XML Treatment for Ulomimus

XML Treatment for Ulomimus indicus
